# Exploring Spanish health social media for detecting drug effects

**DOI:** 10.1186/1472-6947-15-S2-S6

**Published:** 2015-06-15

**Authors:** Isabel Segura-Bedmar, Paloma Martínez, Ricardo Revert, Julián Moreno-Schneider

**Affiliations:** 1 Computer Science Department, University Carlos III of Madrid, Leganés, Madrid, Spain

## Abstract

**Background:**

Adverse Drug reactions (ADR) cause a high number of deaths among hospitalized patients in developed countries. Major drug agencies have devoted a great interest in the early detection of ADRs due to their high incidence and increasing health care costs. Reporting systems are available in order for both healthcare professionals and patients to alert about possible ADRs. However, several studies have shown that these adverse events are underestimated. Our hypothesis is that health social networks could be a significant information source for the early detection of ADRs as well as of new drug indications.

**Methods:**

In this work we present a system for detecting drug effects (which include both adverse drug reactions as well as drug indications) from user posts extracted from a Spanish health forum. Texts were processed using MeaningCloud, a multilingual text analysis engine, to identify drugs and effects. In addition, we developed the first Spanish database storing drugs as well as their effects automatically built from drug package inserts gathered from online websites. We then applied a distant-supervision method using the database on a collection of 84,000 messages in order to extract the relations between drugs and their effects. To classify the relation instances, we used a kernel method based only on shallow linguistic information of the sentences.

**Results:**

Regarding Relation Extraction of drugs and their effects, the distant supervision approach achieved a recall of 0.59 and a precision of 0.48.

**Conclusions:**

The task of extracting relations between drugs and their effects from social media is a complex challenge due to the characteristics of social media texts. These texts, typically posts or tweets, usually contain many grammatical errors and spelling mistakes. Moreover, patients use lay terminology to refer to diseases, symptoms and indications that is not usually included in lexical resources in languages other than English.

## Background

It is well-known that adverse drug reactions (ADRs) are a prominent health matter, being the fourth cause of demise in hospitalized patients [1]. Thus, the field of pharmacovigilance has received a great deal of attention due to the elevated and growing impact of drug safety events [2] as well as their high associated costs [3].

Since many ADRs are not captured during clinical trials, the major drug regulatory organizations, for example, the US Food and Drug Administration (FDA) or the European Medicines Agency (EMA) require healthcare practitioners to inform all suspect adverse drug reactions. However, some investigations have revealed that ADRs are under-estimated due to the fact that they are reported by voluntary reporting systems [3-5]. In fact, it is estimated that only between 2 and 10 per cent of ADRs are reported [6]. Healthcare professionals must perform many tasks during their workdays and thus it is very difficult finding the time to use these surveillance reporting systems. Also, healthcare specialists actually report only those ADRs on which they have absolute firm conviction of their existence. Several medicine agencies have implemented spontaneous patient reporting systems in order to report ADRs by themselves. Some of these systems are the MedWatch from the FDA (http://www.fda.gov/Safety/MedWatch/default.htm), the Yellow Cards from the UK Medicines agency (MHRA) (https://yellowcard.mhra.gov.uk/) or the website (https://www.notificaram.es/) developed by the Spanish Agency of Medicines and Medical devices (AEMPS). Patient reports frequently provide more precise and clear information about ADRs than reports from doctors [7]. Another important contribution of spontaneous patient reporting systems is to encourage patients to be more active in their treatments. However, despite the fact that these systems are well-established, the rate of spontaneous patient reporting is very reduced, presumably due to the fact that many patients have no knowledge about their existence and may even feel troubled when explaining their symptoms.

In this study, our hypothesis is that health social networks could be used as a additional data source to spontaneous reporting systems in order to detect unknown ADRs and thereby to increase drug safety. Currently, social media on health information, just like has happened in other areas, have experienced a huge growth [8]. Examples of social media sites include blogs, online forums, social networks, and wikis, among many others. In this work, we focus on health forums where patients often exchange information about their personal medical experiences with other patients who suffer the same illness or receive similar treatment. Some patients may feel more comfortable sharing their medical experiences with each other rather than with their clinicians. This may happen because patients may feel closer to people with similar problems rather than doctors. These forums include a high number of posts depicting patient experiences that would be a rich source of data to detect unknown ADRs as well as new drug uses.

Although there have been several research efforts devoted to developing systems for extracting ADRs from social media, all studies have focused on social media in English, and none of them have addressed the extraction from Spanish social media. Moreover, although several annotated corpora for ADRs have been created [9,10], none of them consists of Spanish texts from social media. Thus, the comparison of the main systems for ADR extraction from social media has not been possible due to the absence of a gold-standard corpus of social media texts. Therefore, it is very difficult to establish the present "state-of-art" of the techniques for ADR extraction from social media.

Thus, the goal of our work is twofold: i) to create a gold-standard corpus of Spanish social media messages, which are annotated with drugs and their effects and ii) to develop a system to automatically extract drugs and their effects (ADRs as well as drug indications) from Spanish health-related social media sites. The corpus is composed by patients' comments from ForumClínic (http://www.forumclinic.org), a health online networking website in Spanish. This is the former corpus of patient posts annotated with drugs and their effects in Spanish. Also, we believe that this corpus is a good starting point that will facilitate comparison for future ADR detection from Spanish social media. We expect this system will be useful to *AEMPS *as well as to the pharmaceutical industry in the enhancement of their pharmacovigilance technology.

## Related Work

The application of Natural Language Processing (NLP) techniques to mine ADRs from texts has been recently considered with encouraging results, mostly in the issue of drug product labels [11-13], biomedical literature [14], medical case reports [15] and health records [16,17]. Nevertheless, as it will be explained below, ADRs detection from social media has received limited attention.

In general, medical literature, such as scientific publications and drug labels, contain few grammatical and spelling mistakes. Another important advantage is that such texts can be easily linked to biomedical ontologies by matching detected entities to concepts in these semantic resources. Meanwhile social media texts are markedly different from medical literature, and thereby the processing of social media texts poses additional challenges such as the management of metadata associated to the text (such as tags in tweets) [18], the detection of typos and unconventional spelling, word shortenings [19,20], slang and emoticons [21] and lack of punctuation marks, among others. Moreover, these texts are often very short and with an informal nature, making the processing task extremely challenging.

Regarding the identification of drug names in text, during the last four years there have been significant research efforts directed to encourage the development of systems for detecting these entities. Concretely, shared tasks such as DDIExtraction 2013 [22], CHEMDNER 2013 [23] or the i2b2 Medication Extraction challenge [24] have been held for the progress of the state of the art in this area. However, the main body of the work on recognizing drugs concerns either biomedical literature (for example, MedLine articles) or clinical records, without considering social media streams.

Leaman et al. [25] implemented a system to automatically detect adverse effects in user posts. A corpus of 3,600 comments from the DailyStrength site was collected and annotated by hand with a total of 1,866 drug conditions, including beneficial effects, adverse effects, indications and others. To identify the adverse effects in the user comments, a lexicon was compiled from the following resources: (1) the COSTART vocabulary (National Library of Medicine, 2008), (2) the SIDER database [13], (3) MedEffect (http://www.hc-sc.gc.ca/dhp-mps/medeff/index-eng.php) and (4) a list of colloquial sentences which were manually collected from the DailyStrength comments. The final lexicon consisted of 4,201 concepts (terms with the same CUI were grouped in the same concept). Finally, the terms in the lexicon were mapped against user comments to identify the adverse effects. To discriminate adverse effects from the other drug conditions (beneficial effects, indications and others), the system used a list of verbs denoting indications (for example *help, work, prescribe*). Drug name recognition was not necessary because the evaluation focused only on a set of four drugs: *carbamazepine, olanzapine, trazodone *and *ziprasidone*. The system achieved a good performance, with a precision of 78.3% and a recall of 69.9%.

Later, Nikfarjam and Gonzalez [26] extended the above work by applying association rule mining to detect prevalent patterns concerning opinions about drugs. The rules were extracted using the Apriori tool (http://www.borgelt.net/apriori.html), an implementation of the Apriori algorithm [27]. The system was evaluated using the same corpus created for their previous work [25], and which has been described above. The system had a precision of 70.01% and a recall of 66.32%. The major convenience of the system is that it could be easily portable to other domains and languages. Another important advantage of this approach over a dictionary-based method is its capacity to recognize terms not found in the dictionaries.

Benton et al. [28] created a corpus of messages from several online forums about breast cancer, which later was used to extract potential ADRs from the most widely used drugs for this disease: *anastrozole, axemestane, letrozole *and *tamoxifen*. A lexicon of lay medical terms was gathered from drug databases and several websites with information about medicines and their main ADRs. The lexicon was extended with the Consumer Health Vocabulary (CHV) (http://consumerhealthvocab.org), a patient-oriented terminology. Then, pairs of terms that occur in a window of 20 tokens were considered. The Fisher's exact test [29] was applied to determine the probability that the two terms appear in the same window by chance alone. Evaluation was only performed on the four drugs mentioned above. To this, the authors collected their adverse effects from their drug labels. Then, the adverse effects from drug labels were compared with the adverse effects provided by the system. The system obtained an average precision of 77% and an average recall of 35.1% for all four drugs.

Bian et al. [30] developed a system to detect tweets describing ADRs. The systems used a SVM classifier trained on a collection of tweets, which were labelled by two experts. MetaMap [31] was used to analyse the tweets and to find the UMLS concepts present in the tweets. The system produced poor results, mainly because tweets are riddled with spelling and grammar mistakes. Moreover, MetaMap is not a suitable tool to analyse this type of texts since patients do not usually use medical terminology to describe their medical experiences. MetaMap is based on UMLS and other integrated resources such as SNOMED and MedDRA, which do not contain patient oriented vocabulary.

As already mentioned, very few systems have been developed to recognize drugs from social media texts. As well, the extraction of relations between drugs and their effects has hardly addressed, since most efforts have been concentred on the detection of the effects of a given set of drugs. Dictionary-based systems for extracting ADRs fail to identify terms, which are not found in the dictionaries. In addition, these systems also fail to handle spelling and grammar errors, so prevalent in social media texts. Moreover, all systems developed so far have focused on English texts. Indeed, very little research has been done on automatic information extraction from Spanish-language social media in the biomedical domain. Additionally, to the best of our knowledge, there is no corpus annotated with ADRs in Spanish social media texts available today.

## Resources and tools

In [32] Segura-Bedmar et al. presented the first system capable of detecting drugs and their effects from social media channels in Spanish language. It is based on the MeaningCloud tool (http://www.meaningcloud.com/), a commercial tool for Named Entity Recognition (NER), which follows a dictionary-based approach. To create the dictionary, the terms were gathered from several domain resources: CIMA (http://www.aemps.gob.es/cima/) and MedDRA (http://www.meddra.org/). A detailed description of these resources can be found in [32]. Besides, the system also used drug and effect gazetteers derived from websites such as Vademecum (http://www.vademecum.es/), MedLinePlus (http://www.nlm.nih.gov/medlineplus/spanish/) and the ATC system (http://www.whocc.no/atc_ddd_index/), a classification system of drugs. To evaluate the system, the SpanishADR corpus was created. This is the first corpus including Spanish social media texts annotated with drugs and effects. This corpus was annotated by two annotators and consisted of 400 user messages collected from ForumClinic. The inter-annotator agreement (IAA) study showed high agreement for drugs and moderate agreement for effects. The size of the corpus is 26,519 tokens, whereas each message contains an average of 3.15 annotations (0.48 drugs, 1.42 effects and 1.25 relations). More information about the SpanishADR corpus can be found in [32]. Regarding the results, the system showed a precision of 87% for drugs and 85% for effects, and a recall of 80% for drugs and 56% for effects. It should be noted, however, that this system does not support the detection of relations between drugs and their effects.

Recently, we have reported an extension of this system to detect relations between drugs and their effects [33]. Thus, this extended system recognizes drugs and effects, and also extracts relations among them. The system obtains the drug-effect pairs that occur in the same context and then uses a database with information about drugs and their effects (indications and ADRs) to identify those pairs that are related. This database, called SpanishDrugEffectDB, was automatically built from several websites such as MedLinePlus, http://www.prospectos.net/ and http://prospectos.es, which contain a huge number of drug package leaflets. These documents include sections describing the indications as well as ADR for a certain drug. These indications and ADRs were automatically extracted from the documents to populate the database. The SpanishDrugEffectDB database can be a useful resource to automatically identify drug indications and ADRs from texts. Moreover, this is an important contribution since, despite there are various English databases such as SIDER or MedEffect about drugs and their ADRs, SpanishDrugEffectDB is the first database in the Spanish language, which contains this type of information. The reader can find a detailed description of the database as well as its construction process in [33]. The database contains a total of 7,378 drugs, 52,199 effects, 4,877 drug-indication relations and 58,633 drug-ADR relations. To evaluate the system, the SpanishADR corpus was also annotated with the relations between drugs and their effects (drug indications as well as ADRs). The results of the evaluation showed that the system achieved very high precision (83%) but that recall was much lower (15%).

## Methods

In general, co-occurrence systems provide high recall but low precision rates. It is well known that Supervised Machine Learning methods produce the best results in Information Extraction tasks. One major limitation of these methods is that they require a significant number of annotated training examples. Unfortunately, there are very few annotated corpora because their construction is costly.

In this paper, we propose a system based on distant supervision [34], an alternative solution that does not need annotated data. The distant supervision hypothesis establishes that if two entities occur in a sentence, then both entities might participate in a relation. The learning process is supervised by a database, rather than by annotated texts. Thus, this approach does not imply overfitting problems that produce a domain-dependence in almost all supervised systems.

Thus, while in a classical supervised approach the system would be limited to the small size of the SpanishADR corpus, which only contains 400 user messages [32,33], the main advantage is that a system based on distant supervision can use all user messages (84,090) collected from ForumClínic. In particular, our system uses the SpanishDrugEffectDB database to train a distant supervision model for relation extraction.

### Creating training and testing data

**Figure 1:**
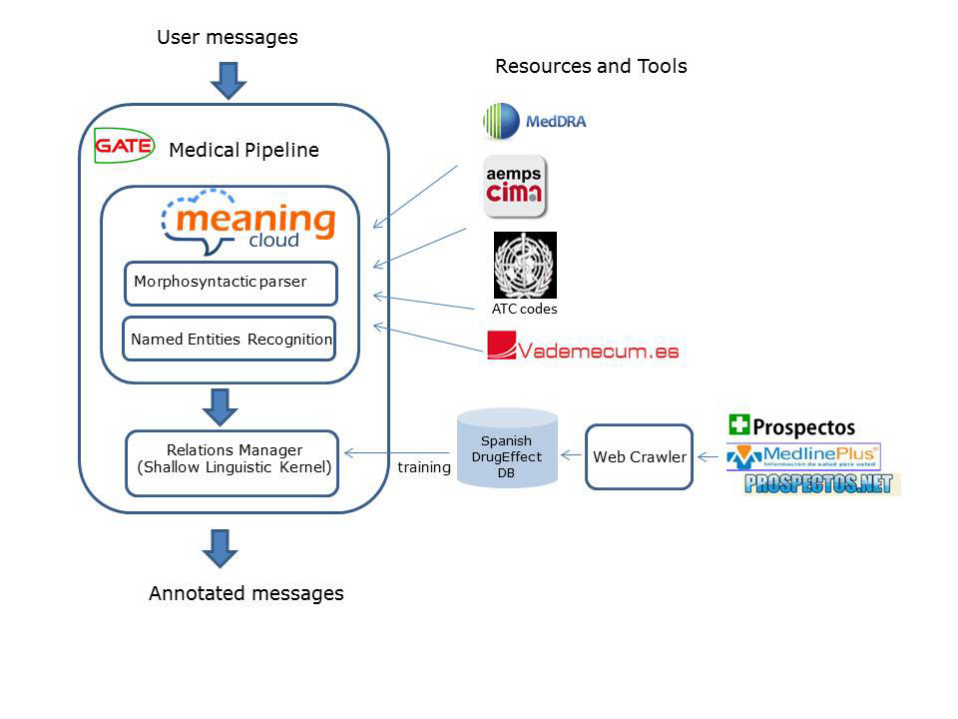
**Pipeline integrated in GATE platform to process user messages**.

Firstly, all user messages from ForumClínic were processed using our GATE pipeline (see Figure 1) to identify the mentions of drugs and their effects in texts. The reader can find a detailed description of this pipeline in [32,33]. Once entities were automatically annotated, all pairs (drug, effect) which occurred in a window size of 250 tokens were considered as relation instances. Each relation instance was searched for in the SpanishDrugEffectDB database in order to know if it is a positive instance. Then, the collection of user messages was split into two different datasets, leaving 75% for training (with a total of 63,067 messages) and 25% (21,023 messages) for testing. In this way, the database gives us a training set of relation instances to train any supervised algorithm.

### Shallow Linguistic Kernel

We decided to use the Shallow Linguistic (SL) kernel proposed by Giuliano et al. [35] because it has been shown to perform well using only shallow linguistic features. Furthemore, we think that kernel methods incorporating syntactic information are not suitable for social media texts, since many sentences are ungrammatical, and thereby, a syntactic parser is not able to correctly process them. Another important advantage is that the performance of the SL kernel does not seem to be influenced by named entity recognition errors [36]. The SL kernel is a linear combination of two sequence kernels, Global Context and Local Context.

The global context kernel is able to recognize the existence of a binary relation using the tokens of the entire sentence. Bunescu and Mooney [37] claim that binary relations are characterized by the tokens that occur in one of these contexts: Fore-Between (FB), Between (B) or Between-After (BA). As it is well known in Information Retrieval, stop-words and punctuation marks are usually removed because they are not useful to find documents. However, these features are valuable clues for identifying relations. For this reason, they are preserved in the contexts. The similarity between two relation instances is calculated using the n-gram kernel [38]. For each of the three contexts (FB, B, BA), an n-gram kernel is defined by counting the common n-grams that both relation instances share. Finally, the global context kernel is defined as the linear combination of these three n-grams kernels.

The local context kernel is able to determine if two entities are participating in a relation by using the context information associated with each entity. Thus, two windows around the entities are considered: the left local context for the first entity that appears in the sentence and the right local context for the second one. Lexical and morphological features such as tokens, lemmas, Part-of-Speech (PoS) tags and stems are used to represent each local context. The local context kernel is defined as the sum of the left context kernel and the right context kernel. The main difference between the local and the global context kernel is that the local one uses morphological features (such as PoS tags, lemmas and stems) whereas the global one does not. The reader can find a detailed description of both kernels in [35].

## Results and discussion

As mentioned above, the collection of user messages was randomly split 75% for training and 25% for testing for the Relation Extraction task. Then, the database was used to label each relation instance as a positive or negative instance in both datasets. We are aware that this type of automatic evaluation suffers from false negatives, but it provides a realistic estimation of precision without requiring expensive manual evaluation.

Table 1 shows the results of the SL kernel on the testing dataset and on the SpanishADR corpus.

**Table 1:** Results of the distant supervision method.

Evaluation Dataset	TP	FP	FN	Precision	Recall	F1
Test (25%)	1,755	1,926	1,224	0.48	0.59	0.53
Gold Standard	41	27	123	0.60	0.25	0.35

A previous work described in [33], which combined a co-occurrence method with the SpanishDrugEffectDB database, showed a precision of 83% and a recall of 15%. The distant supervision method presented here was evaluated on the SpanishADR corpus and achieved an improvement of 10% in recall at the expense of a decrease of 23% in precision (see second row of Table 1) compared to our previous system.

The distant supervision method produces an important number of false negatives because the SpanishDrugEffectDB database is incomplete. On the other hand, it should be noted that this database was automatically built without human review, and this could be the main cause of the false positives generated by the method. Therefore, it is expected that an improvement in the quality of the database may lead to a significant improvement in the results yielded by the distant supervision method.

Furthermore, we performed a detailed error analysis with the objective of providing a road map for future work for improving the NER issues as well as in the extraction of drug-effect relations. To this end, we focus on the study of the main source of errors produced by the system developed. We will first introduce the errors regarding the NER, followed by a complete analysis of the Relation Extraction problems.

On the one hand, concerning the NER task, a sample of 50 user messages was randomly selected and analysed. Regarding the detection of effects, the major cause of false negatives was the use of colloquial expressions to describe an effect. Phrases like *me deja ko *(it makes me KO) or *me cuesta más levantarme *(it's harder for me to wake up) were used by patients for expressing how they felt. These phrases are not included in our dictionary. A possible solution may be to create a lexicon containing these lay expressions. The second highest cause of false negatives for effects was due to the different lexical variations of the same effect. For example, *depresión *(depression) is included in our dictionary, but their lexical variations such as *depremido *(depress), *me deprimo *(I get depressed), *depresivo *(depressive) or *deprimente *(depressing) were not detected by our system since they are not in our dictionary. Nominalization may be used to identify all the possible lexical variations of a same effect. Another important error source of false negatives was spelling mistakes (eg. *hemorrajia *instead of *hemorragia)*. Many users have great difficulty in spelling unusual and complex technical terms. This error source may be handled by a more advanced matching method capable of dealing with the spelling error problem. The use of abbreviations (*depre *is an abbreviation for *depresión*) also produces false negatives. Linguistic pre-processing techniques such as lemmatization and stemming may help to handle this kind of abbreviations.

The main source of false negatives for drugs seems to be that users often misspelled drug names. Some generic and brand drugs have complex names for patients. Some examples of misspelled drugs are *avilify *(*Abilify*) or *rivotril (Ribotril)*. Another important source of errors was the abbreviations for drug families. For instance, *benzodiacepinas *(benzodiazepine) is commonly used as *benzos*, which is not included in our dictionary. An interesting source of errors to point out is the use of acronyms referring to a combination of two or more drugs. For instance, FEC is a combination of *Fluorouracil, Epirubicin *and *Cyclophosphamide*, three chemotherapy drugs used to treat breast cancer.

Most false positives for drugs were due to a lack of ambiguity resolution. Some drug names are common Spanish words such as *Allí *(a slimming drug) or *Puntual *(a laxative). Similarly, some drug names such as *alcohol *(alcohol) or *oxígeno *(oxygen) can take a meaning different than the one of pharmaceutical substance. Another important cause of false positives is due to the use of drug family names as adjectives that specify an effect. This is the case of *sedante *(sedative) or *antidepresivo *(antidepressant), which can refer to a family of drugs, but also to the definition of an effect or disorder caused by a drug (sedative effects).

On the other hand, regarding the Relation Extraction task, we randomly selected a sample of 1506 comments from the test dataset (approximately 7% of it). In order to know the volume of messages reporting about treatments, in a first analysis messages were classified according to the their annotations: messages having nor drug neither effect (55%), messages without a drug (27%), messages without an effect (5%) and messages with drug(s) and effect(s) annotated (13%). This means that approximately half of them are not related to drug treatments.

Regarding the false positives (see Table 2), the main source of errors is the lack of context resolution. This means that, despite correctly detecting a drug and an effect (according to the drug package insert), the context of the text did not fulfill the requirements to properly consider it a relation. In the example **FP1 **(see Table 3) we can see how *diabetes *and *Escitalopram *are considered a pair by the system, despite the fact that the user is talking about them in two different contexts. Moreover, in **FP2 **(see Table 3) we can see how the lack of co-reference resolution introduces another important source of error for false positives. The user introduces the term *side effects *and then talks about two of them in particular. This kind of cataphora is not correctly solved by the system. Another case of false positives is due to the fact that either the drug or the effect needs a modifier in order for the phrase to acquire complete meaning. In **FP3 **(see Table 3) the user is talking about *lithium poisoning*, and introducing the effects it may cause. In the system, these effects are attributed to *lithium *itself, instead of to the poisoning caused by it. In this case, depending on the dosage of the drug, the relations cannot be considered correct. An interesting source of errors is the lack of negation resolution, which means that even though the user specifies he/she did not experience an effect after taking a drug, the system annotates the relation. In **FP4 **(see Table 3) we can see how a user expresses happiness for not having experienced *hot flashes *after taking *Xeloda*. Finally, the complex sentences (coordinated and subordinated sentences) in a comment may mislead the system into annotating a relation which is not correct, giving place to another interesting source of false positives. For instance, in the example **FP5 **(see Table 3) the relation between *schizophrenia *and *Anafranil *was annotated. As we can see, there are three effects one after the other, and a drug which is separated from them with an intermediate phrase where the user gives his/her opinion about the topic they are discussing. Furthermore, in the *Anafranil's *drug package insert it is pointed out that patients with *schizophrenia *should be cautious if taking the drug, but it does not say that this effect is an indication or an adverse effect for this drug.

**Table 2:** Analysis of false positives in the test dataset.

Error cause	False Positives	Examples
Different context	62	FP1
Co-reference resolution required	46	FP2
Modifier needed for full understanding	28	FP3
Lack of negation resolution	13	FP4
Syntactically Complex phrases	9	FP5
Total	158	

**Table 3:** Example of false positives in the test dataset.

ID	Example	FP
FP1	*Lo del aumento del azúcar lo decía porque tengo en mi familia antecedentes de **diabetes_e1 _**y me da **miedo_e2 _**que este medicamento pueda a la larga generarme esta **enfermedad_e3_**. Otra cuestión... Por las mañanas tomo **Escitalopram_d1_**, que me pone muy nerviosa*.	(d1, e1)
FP2	*Cada vez que voy a por las pastillas de **Xeloda_d1 _**me siento con el farmacéutico y me pregunta qué tal con los **efectos secundarios_e1_**, la primera vez me dijo que los más comunes son la **diarrea_e2 _**y las **rojeces_e3_***	(d1,e1)
FP3	*La **intoxicación con litio_d1 _**produce los siguientes síntomas: **náuseas_e1 _**o **malestar digestivo_e2 _**importante, **vómitos_e3_**, **temblor de manos**_e4 _acrecentado, **diarrea_e5_**, **problemas de memoria_e6_**, **visión borrosa_e7 _**y **dificultades de coordinación de movimientos_e8_***	(d1,e1),(d1,e2), (d1,e3),(d1,e4),(d1,e5),(d1,e6), (d1, e8)
FP4	*También tomo **Xeloda_d1_**, en mi caso llevo 5 ciclos de 6, no tengo ningún **efecto secundario_e1_**, todo perfecto (que alegría que no tengo **sofocos_e2_**)*.	(d1, e2)
FP5	*Sólo hay tres enfermedades mentales: **depresión mayor_e1_**, **depresión ansiosa_e2 _**y **esquizofrenia_e3_**. Lo demás es marketing para vender más medicamentos. De todas formas, mientras te mantengan el **Anafranil_d1 _**seguirás estando bien*.	(d1, e3)

With respect to the false negatives (see Table 4), the major source of errors is the long distance between the pair drug-effect in the text. In **FN1 **(see Table 5) we have an example where a drug and an effect are not annotated as a relation because the system does not consider it due to this problem. The syntactic complexity of a comment is another source of false negatives. In the example **FN2 **(see Table 5) the system is misled because of the phrase structure of the comment, in this case, a coordinate structure. Moreover, we have also realized that very simple phrases such as **FN3 **(see Table 5) may also bring confusion. As we can see, the simplicity of the comment (and adjective plus a prepositional phrase) caused a false negative in this example; perhaps in this case the SL model did not learn this kind of syntactic structures if the training dataset had few examples of them. Furthermore, as it happens with false positives, the need of a modifier to give a complete meaning to a drug or an effect is also a source of error for false negatives. We can observe in **FN4 **(see Table 5) how the system could not annotate the *heat *illness as it could not understand whether the body heat increased or decreased. As a matter of fact, the relation between *Tamoxifen *and the effect was not successfully annotated. Finally, the lack of co-reference resolution is the last source of errors. As we can see in **FN5 **(see Table 5) the effect *e1 *is related to the drug *d2*, but *e2*, which is an anaphora of *e1 *is not related to *d2*.

**Table 4:** Analysis of false negatives in the test dataset.

Error cause	False Negatives	Examples
Long distance between pair entities	254	FN1
Syntactically Complex phrases	76	FN2
Simple phrases	14	FN3
Modifier needed for full understanding	10	FN4
Co-reference resolution required	6	FN5
Total	414	

**Table 5:** Example of false negatives in the test dataset.

ID	Example	Relations not detected
FN1	*La respuesta al tratamiento biológico en **espondilitis anquilosante_e1 _**suele ser buena y rápida. [⋯] Más del 70% de los pacientes mejoran mucho con los biológicos, indistintamente de cuál se utilice. Dicen que **Infliximab_d1 _**es algo más potente*.	(d1,e1)
FN2	*Tendré que raparme al acabar la **quimio_d1 _**para que ya empiece a nacer en condiciones, y por lo demás, el **cansancio_e1_**, y el **sabor metálico en la boca_e2_**, **dolor muscular_e3_**⋯ nada nuevo*.	(d1,e1),(d1,e2)
FN3	*Soy **adicto_e1 _**a las **benzodiacepinas_d1_***.	(d1,e1)
FN4	*Si puedes me cuentas como te fue y los **efectos_e1 _**que tienes con el **Tamoxifeno_d1_**, ya que yo paso de tener frio a tener **mucho calor_e2_***.	(d1,e2)
FN5	*Los **antipsicóticos_d1 _**no crean **dependencia_e1_**, los únicos fármacos que crean este **problema_e2 _**si no se toman adecuadamente son las **benzodiacepinas_d2_***.	(d2,e2)

## Conclusions

The final goal of our research is the detection of ADRs and drug indications from social media texts. Most systems for detecting drug effects from texts use simple dictionary based methods to recognize the entities and pattern-based approaches to extract the relations between them. The lack of annotated corpora complicates the application of supervised learning methods. On the contrary, distant supervision is a learning approach where the classifier algorithm is supervised by a knowledge base rather than by an annotated corpus. The major benefit of using distant supervision is that this paradigm does not need annotated corpora, which is usually costly to create. Due to this fact, distant supervision has generated considerable interest for training relation extraction systems [34,39,40]. Results of such systems are promising. In this paper we present a system based on the distant supervision paradigm to detect drug effects (ADRs and drug indications) from user messages, which were collected from a Spanish health website. To the best of our knowledge, our work is the first system that applies a distant supervision approach to solve this problem. Unfortunately, our results cannot be compared with those obtained for the previously introduced systems because they treat other types of relations and other types of texts.

It should be noted that social media texts pose new challenges that are not present in the processing of medical literature. These new problems are the management of metadata included in the text [18], the detection of misspellings, word shortenings [19,20], slang and emoticons and to cope with ungrammatical phrases, among others. Moreover, while many terms present in clinical records and medical literature could be linked to domain resources, lay terms are not usually recorded in any structured resource. This lack of lay vocabularies makes difficult the automatic processing of social media texts. Another important challenge is the lack of appropriate resources, such as comprehensive knowledge bases on drugs and their effects as well as annotated corpora from social media texts, to be used in this task.

Taking into account the great challenges of the processing of social media texts, we can conclude that our system provides very encouraging results. As mentioned above, the precision of the distant supervision could be improved if we manually review the database in order to remove false positives, which were generated by the automatic process used to build the database. The major advantage of the distant supervision paradigm is that it does not need any manual annotation. In contrast, this paradigm is negatively affected by the incompleteness of the knowledge bases used. Thus, if our database could be augmented from other websites about drugs and their effects, the recall may also be increased.

## Competing interests

Authors have no competing interests.

## Authors' contributions

**ISB **designed the study, developed the system and performed the experiments. She also participated in the annotation of the corpus. PMF contributed with ideas and helped in the design of the SpanishDrugEffectDB and in the annotation of the corpus. RR and JMS participated in the development of experiments. This document was prepared by ISB and PMF.

## Declarations

The authors declare that funding for publication of the article was sponsored by European Commission under the Seventh Framework Program, contract No. 287863

This article has been published as part of *BMC Medical Informatics and Decision Making *Volume 15 Supplement 2, 2015: Proceedings of Louhi 2014: The Fifth International Workshop on Health Text Mining and Information Analysis. The full contents of the supplement are available online at http://www.biomedcentral.com/bmcmedinformdecismak/supplements/15/S2.
